# Challenges of a Pregnant Woman With Untreated Myasthenia Gravis: Delivery and Puerperium

**DOI:** 10.7759/cureus.86517

**Published:** 2025-06-22

**Authors:** Marta Plancha, Andreia Miguel, Francisca Magno, Margarida Silva Dias, Ana Isabel Machado

**Affiliations:** 1 Obstetrics and Gynecology, Unidade de Saúde Local de São José, Maternidade Dr. Alfredo da Costa, Lisboa, PRT; 2 Neurology, Unidade de Saúde Local de São José, Hospital de São José, Lisboa, PRT; 3 Obstetrics and Gynecology, Faculdade de Medicina da Universidade Nova de Lisboa, Nova Medical School, Lisboa, PRT

**Keywords:** contraception, labor, myasthenia gravis, pregnancy, puerperium

## Abstract

Myasthenia gravis (MG) is a rare autoimmune neurological disorder. Pregnancy, labor, and puerperium are risk factors for a myasthenic crisis. This case reports a late 20s pregnant woman evacuated from Guinea-Bissau with the diagnosis of generalized and severe MG, tetraparetic, at 38 weeks of gestation. She had never been treated for MG. After multidisciplinary assessment and despite current recommendations, intensive therapy with IV immunoglobulin and pyridostigmine was started and labor was induced. There were no neonatal complications. There was a worsening of symptoms during puerperium and therapy intensification with additional corticosteroids being needed.

Labor in women with non-treated MG carries a high risk for serious and even fatal complications both for mother and child. Despite being a rare condition, it is more frequent in women of childbearing age, and guidelines on this topic are scarce.

## Introduction

Myasthenia gravis (MG) is a chronic autoimmune disorder of the neuromuscular junction, mediated primarily by antibodies against the acetylcholine receptor (AChR), leading to fatigable muscle weakness. It is twice as common in females, with a prevalence between 1:10.000 and 1:50.000. Most cases are diagnosed before the age of 30 which increases the relative likelihood of its occurrence during pregnancy and postpartum [1-8].

MG may be due to blockage, alteration, or destruction of AChR [1-3,9]. There are three known forms of the disease: a purely ocular form, a generalized form, and a bulbar dominant presentation. In the generalized form, weakness may involve oropharyngeal, respiratory, and skeletal proximal muscles. Bulbar symptoms can also be present with the appearance of dysarthria, dysphagia, and nasal voice. AChR antibodies are present in 85% of patients with generalized MG. The bulbar dominant form primarily affects muscles of the face, throat, and neck, leading to prominent dysarthria, dysphagia, nasal voice, and chewing difficulties, often without significant limb weakness. Respiratory compromise may occur if progression ensues. In the ocular form, weakness affects the periocular muscles, causing fluctuating ptosis and diplopia and 40-60% of the patients have AChR antibodies. A subset of AChR-negative cases harbor antibodies against muscle-specific kinase (MuSK; ∼30-40% of AChR negative) or low-density lipoprotein receptor-related protein 4 (LRP4; ∼5-10% of AChR negative). The remaining patients without detectable antibodies to AChR, MuSK, or LRP4 are classified as triple-seronegative [1-6,9,10]. MG evolves in flare-ups/remissions. Several factors can lead to an exacerbation of the disease, such as physical and emotional stress, infections, thyroid disorders, surgery, general anesthesia, certain medications, menses, pregnancy, labor, and puerperium [7,9,10-12]. The myasthenic crisis is characterized by severe generalized muscle weakness, including weakness of the respiratory muscles, leading to respiratory failure which may require intensive care unit admission and mechanical ventilation [4,9].

The course of MG during pregnancy is unpredictable [2,7,13]. Some studies indicate that symptoms worsen during pregnancy in 30% of women, remain unchanged in 30 to 40% and may even improve in the remaining 20 to 30% [1-5,8,10,13,14]. Regarding the timing of exacerbation, it occurs more often during the first trimester and in the puerperium [2,3,8,9,10,11,15]. However, a deterioration of ventilatory function requiring mechanical ventilation has also been described in approximately 20% of pregnant women with MG at some point during pregnancy [4]. The improvement of symptoms during pregnancy, particularly in the second trimester, may be related to rising levels of alpha-fetoprotein (AFP). This glycoprotein peaks between 15 to 20 weeks’ gestation and appears protective in MG due to its inhibitory effect on anti-AChR antibodies. Conversely, symptom exacerbation postpartum correlates with the rapid decline in AFP levels after delivery [8,11,12,14,15]. Recommendations vary: while some advise a two- to three-year delay post-diagnosis, contemporary guidelines prioritize more than six to 12 months of stable disease (minimal symptoms on low-risk therapies) and absence of bulbar/respiratory involvement. Uncontrolled MG increases exacerbation risk during pregnancy up to 40% [1,2,4,8,10]. In most studies, there is no increased risk of miscarriage, preterm birth, intrauterine growth restriction, hypertensive disorders, or cesarean with MG [1,9,15-17]. Although hypertensive disorders do not seem to be increased in MG, the management and prevention of seizures in pre-eclampsia and hemolysis, elevated liver enzymes, low platelet count (HELLP) syndrome are affected, as magnesium sulfate, which reduces ACh release, is contraindicated because it can promote a myasthenic crisis [1,9,10,12,14,15]. As an alternative, phenobarbital, phenytoin or diazepam can be administered [1].

Labor can be a triggering factor for a myasthenic crisis. According to current recommendations in women clinically stable or well-controlled, the timing and type of delivery should not be influenced by MG, and should be conditioned by obstetric indications solely [2,4,7-9,13,15]. As uterine smooth muscle is not compromised, the first stage of labor is usually not affected. In contrast, the second stage, which relies heavily on voluntary muscles, may be prolonged, increasing the likelihood of requiring an instrumented delivery [2,4,8,9,13,15]. Also, there are precautions to be taken with anesthesia, as narcotics and sedatives can trigger respiratory depression in women with MG [3,8,9,14,17]. Epidural analgesia has no contraindications and is recommended to reduce the need for systemic medications [4,8,13,17]. The puerperium is a period of vulnerability, and intensive care surveillance is advised since approximately one-third of patients may have a myasthenic crisis [11,12,14,16,17]. Ten to 20% of the newborns of mothers with MG may develop neonatal MG due to the transplacental passage of antibodies against AChR [1-3,4,9,13,15,16]. It is characterized by generalized hypotonia, poor sucking, weak cry, and respiratory difficulties, which may require admission of the child in an intensive care unit [1,4,9,16]. Symptoms typically appear within hours of birth (range: 1-72 hours), with complete recovery usually occurring within two to four weeks with appropriate treatment [2].

Thymectomy is recommended in women <60 years with generalized MG due to its disease-modifying effect: it promotes immunological remission, reduces long-term immunosuppression needs, and improves clinical outcomes, even in thymoma-free cases [2,8,18]. In women in whom the diagnosis is made during pregnancy, it is recommended to postpone investigation with CT and surgery a few months after delivery [2,4,8,12]. Oral pyridostigmine is the first-line treatment in pregnant women with MG. In case of exacerbation, corticosteroids, intravenous immunoglobulin (IVIG) and plasma exchange can be used as well [4,7,9,12,14,15,18]. There are no studies to recommend the use of monoclonal antibodies during pregnancy.

Herein we report a case of a woman with myasthenic crisis at 38 weeks of gestation where a concerted involvement of neurologist, obstetrician, anesthesiologist, pediatricians, and intensive care resulted in an uneventful outcome both for the mother and child. As no published guidelines exist on this topic, sharing individual experiences is most relevant.

## Case presentation

A late 20s primigravida was evacuated from an African country with the presumptive diagnosis of generalized myasthenia gravis. She had been diagnosed two years before when she presented with a progressive and oscillating picture of diplopia, bilateral eyelid ptosis, dysphagia, nasal regurgitation, weight loss of 20 kg and a lack of strength in all limbs. Head computed tomography was normal and a chest tomography described a thymic hyperplasia or thymoma. Despite the diagnosis of generalized myasthenia gravis, no medication was prescribed. The pregnancy was planned but poorly monitored, and folic acid/iron supplementation was not administered. During the first and second trimesters there was a worsening of limb strength and dysphagia which culminated with evacuation to our maternity at 38 weeks of gestation with a severely sick patient. Physical examination revealed tetraparesis, ptosis, and dysarthria that clearly got worse with fatigue. An obstetric ultrasound was performed showing an active cephalic fetus, normal growth with a fetal weight estimate of 3166 g and the placenta was in the upper posterior position. She was admitted and evaluated by internal medicine, neurology, and anesthesia. The diagnosis of myasthenia gravis was confirmed with positive anti-acetylcholine receptor antibodies and positive fatigability test in a patient with a previous diagnosis. After a multidisciplinary discussion, she was started on intravenous immunoglobulin 25 mg once a day for five days and pyridostigmine 60 mg three to four times a day, with significant and rapid improvement of symptoms. Labor was induced at 39 weeks with vaginal misoprostol due to an unfavorable cervix (Bishop score <6). Epidural anesthesia was administered at 3 cm dilatation, and the patient subsequently achieved an unassisted vaginal delivery. Delivery occurred five hours after taking 50 mcg of misoprostol. Postpartum hemorrhage was managed with 800 mcg rectal misoprostol. The male newborn weighed 3815 g and had an Apgar Score of 9/10. The newborn was transferred to the neonatal care unit and kept under observation for seven days to exclude the presence of neonatal myasthenia gravis. The pediatric assessment was normal throughout the observation.

Within two hours postpartum, the mother exhibited symptom worsening with decreased strength, bilateral eyelid ptosis, dysphagia, and dysphonia. There was never respiratory distress. As she was already being treated with intravenous immunoglobulin and pyridostigmine 60 mg twice a day, prednisolone 60 mg/day was added. A rapid improvement was observed 24 hours after the delivery, thus allowing transfer to normal ward.

While MG does not contraindicate any contraceptive method, when providing contraceptive advise, it is important to take into account that chronic high-dose steroid therapy and medroxyprogesterone acetate can have a synergistic effect on bone loss. The patient went to a family planning consultation and chose the placement of a copper intrauterine device. However, it had to be removed later through laparotomy due to migration to posterior vaginal cul de sac. She subsequently transitioned to an etonogestrel subcutaneous implant, achieving amenorrhea with good tolerance.

Two months after delivery, she was diagnosed with pulmonary tuberculosis and was medicated with rifampicin/isoniazid for one year. Thymectomy was performed only one year postpartum as it was necessary to wait for two negative bronchial lavage culture results before surgery. She is now on chronic medication for stable myasthenia gravis: prednisolone 20 mg/day, pyridostigmine 60 mg four times daily, and intravenous immunoglobulin 25 g/month. She awaits approval of rituximab for long-term therapy.

## Discussion

In patients with MG, myasthenic crisis or exacerbation can be triggered by pregnancy and/or labor especially in non-medicated patients. This is a serious condition with increased maternal-fetal morbimortality which requires emergent and intensive therapy taken by a multidisciplinary team [10,15]. In a recently published systematic review by Kumar et al. (2023), the authors emphasize that healthcare providers must be knowledgeable about MG and the interdisciplinary diagnostic and therapeutic care it requires. Although in patients with treated and controlled disease, current guidelines recommend to follow obstetric indications, in contrast, in patients suffering from a myasthenic exacerbation, no guidelines exist. In this patient and after a multidisciplinary discussion, we decided on upfront intensive treatment followed by labor induction. There were no complications for the mother or the newborn.

The diagnosis of MG is based on a typical history and confirmed by several tests as abnormal elevated levels of AChR antibodies and electroneuromyography, and can be made during pregnancy [3-5,13]. In our case report the history and neurological examination were typical of MG, and the diagnosis was easily confirmed with positive AChR antibodies. Due to the concern of radiation during pregnancy, CT scan was only performed after delivery to investigate the presence of thymoma.

The risk of mortality in women with MG seems to be inversely related to its duration, peaking in the first year after diagnosis [4,10,15,17,19]. In our case, the diagnosis of MG was made two years before and no medication had been used, carrying a high risk of flare during pregnancy. A multidisciplinary, coordinated and rapid assessment was necessary in order to control the disease before delivery, a moment that could trigger a myasthenic crisis [12,19]. Concerning treatment, pyridostigmine, an oral medication, is the first-line treatment in women with MG and is used for symptom control. Intravenous immunoglobulin and corticosteroids can be used in pregnancy in order to control an exacerbation [2,5,10,14,15,19]. Intravenous immunoglobulin is also used before surgery and as preparation for women giving birth in order to induce a rapid symptomatic improvement and contribute to a faster recovery. In our patient, we used them to induce an immediate response and as a preventive medication to reduce the risk of a flare in the postpartum period. Importantly, prophylactic intravenous Immunoglobulin and corticosteroids are reserved for high-risk cases, not universally applied. In this patient, prophylaxis was indicated due to bulbar symptoms and steroid dependence [5,10,14,15].

Regarding type of delivery there are no firm recommendations in women with decompensated disease. The decision to induce vaginal delivery followed obstetric guidelines, as there were no indications for cesarean section. Cesarean delivery in MG is reserved for specific obstetric or neurological complications (e.g., myasthenic crisis, acute respiratory compromise, or obstetric emergencies) due to its higher risks of postoperative myasthenic exacerbation, respiratory depression from anesthesia and wound healing complications in immunosuppressed patients. In this case, labor induction allowed delivery during peak of intravenous immunoglobulin efficacy while avoiding surgical risks. Although her Bishop score was <6 (indicating unfavorable cervix), her excellent response to pyridostigmine and intravenous immunoglobulin ensured optimal myasthenic control for vaginal delivery. Combined with her strong motivation, this supported our decision to proceed with labor induction.

Tsurane et al. proposed an innovative approach for management of labor and delivery in patients with myasthenia gravis. Accordingly, our case was classified as Category A and epidural anesthesia, at the onset of labor, and immediate vacuum delivery, once fetal head descended to station +2, were indicated [20]. We performed early epidural analgesia at 3 cm of dilatation because it reduces stress, fatigability and the likelihood of a myasthenic crisis, and it is suitable for operative vaginal delivery or C-section if it was necessary. As in the second stage of labor, striated muscles are used to augment expulsion, the use of assisted delivery may be indicated. In our case, no instrumentation was needed [17].

The risk of postpartum hemorrhage is not increased in women with MG. In our case it was minor and quickly controlled with rectal misoprostol.

As 10 to 20% of the newborns may develop neonatal MG, there was a team of neonatologists that immediately evaluated the newborn after birth [10,19]. As a precaution, he was kept under surveillance at the neonatal intermediate care unit for seven days but never showed any signs of neonatal MG.

As described in previous studies, there was a worsening of symptoms in the postpartum period [11,13,15]. We used an aggressive therapy immediately after delivery and prednisolone 60 mg/day was started in order to prevent the occurrence of a myasthenic crisis with respiratory failure.

As up to two-thirds of women report worsening of MG symptoms with menstruation, some studies recommend the use of contraception with progestins or continuous hormonal contraceptives [2,8]. Despite this counseling, a copper intrauterine device was placed. This can be placed in the first 48 hours after delivery or after four weeks, because when placed earlier there is an increased risk of uterine perforation, expulsion, and infection. Despite having been placed at the proper timing, a perforation of the uterus occurred, leading to the dislodgement of the device. There are no reports showing an increased risk of complications with intrauterine contraception among women with myasthenia gravis [2,8]. As opioids and general anesthetics should be avoided due to risk of potentiating AChR antibody effect, the extraction of copper uterine device was performed by mini laparotomy under spinal anesthesia.

Thymectomy has a well-established role in MG and is recommended, as it improves prognosis and reduces exacerbations. It is recommended that thymectomy be postpone until after pregnancy due to its delayed effect [4,12,15,19]. In this case, it was scheduled a few months after delivery, but the surgery was postponed due to an infectious complication. While tuberculosis remains endemic in some African countries, making coincidental reactivation possible, we speculate that corticosteroid immunosuppression may have contributed to disease activation in this patient. Importantly, intravenous immunoglobulin carries thrombogenic risks - particularly relevant in the hypercoagulable state of late pregnancy and early postpartum period. This required careful thrombosis prophylaxis during her treatment [12].

## Conclusions

This report illustrates that despite high mortality risks associated with untreated myasthenia gravis in pregnancy - including 4-8% maternal mortality and 5-10% perinatal mortality - coordinated multidisciplinary management achieved a successful outcome. The case underscores the urgent need for standardized guidelines addressing this critical population, where uncontrolled MG carries mortality rates three to five times higher than in managed disease. Prompt recognition and specialized interventions are essential to mitigate these severe risks for both mother and newborn.
